# Did border closures slow SARS-CoV-2?

**DOI:** 10.1038/s41598-022-05482-7

**Published:** 2022-02-01

**Authors:** Mary A. Shiraef, Paul Friesen, Lukas Feddern, Mark A. Weiss, COBAP Team, Hawraa Al Janabi, Elizabeth Beling, Jonathan Falcone, Lukas Feddern, Cora Hirst, Nora Murphy, Elizabeth Stifel, Erin Straight, Dongying Tao, Erin Tutaj, Mark A. Weiss, Mary A. Shiraef

**Affiliations:** 1 Department of Political Science, University of Notre Dame, Notre Dame, IN USA; 2 Department of Health Economics and Health Services Research, University Medical Center Hamburg-Eppendorf, Hamburg, Germany; 3 Department of Economics, University of Konstanz, Konstanz, Germany; 4 Departments of Political Science and Mathematics, Emory University, Atlanta, GA USA; 5 Departments of Biological Sciences and Romance Languages & Literatures, University of Notre Dame, Notre Dame, USA; 6 Department of Sociology, Emory University, Atlanta, GA USA; 7 Department of Political Science, University of Waterloo, Waterloo, Canada; 8 Departments of Biology and Anthropology, Emory University, Atlanta, GA USA; 9 Center for Latin American, Caribbean & Latinx Studies, Vanderbilt University, Nashville, TN USA; 10 Department of Leadership, Policy, and Organizations, Vanderbilt University, Nashville, TN USA

**Keywords:** Disease prevention, Immunology

## Abstract

Despite the economic, social, and humanitarian costs of border closures, more than 1000 new international border closures were introduced in response to the 2020–2021 pandemic by nearly every country in the world. The objective of this study was to examine whether these border closures reduced the spread of the severe acute respiratory syndrome coronavirus 2 (SARS-CoV-2). Prior to 2020, the impacts of border closures on disease spread were largely unknown, and their use as a pandemic policy was advised against by international organizations. We tested whether they were helpful in reducing spread by using matching techniques on our hand-coded COVID Border Accountability Project (COBAP) Team database of international closures, converted to a time-series cross-sectional data format. We controlled for national-level internal movement restrictions (domestic lockdowns) using the Oxford COVID-19 Government Response Tracker (OxCGRT) time-series data. We found no evidence in favor of international border closures, whereas we found a strong association between national-level lockdowns and a reduced spread of SARS-CoV-2 cases. More research must be done to evaluate the byproduct effects of closures versus lockdowns as well as the efficacy of other preventative measures introduced at international borders.

## Introduction

This study examined whether the international border closures introduced throughout the 2020–2021 pandemic reduced the spread of SARS-CoV-2. Despite the intuitive link between international travel and a novel virus’ spread, the World Health Organization (WHO) explicitly advised against border closures prior to this pandemic, advocating instead for targeted screening measures and other preventative measures at international borders.^1,2^ Yet, nearly every country in the world introduced a new international border closure in response to the COVID-19 pandemic. We analyze the impact of these border closures on virus spread using the COVID Border Accountability Project (COBAP) database.^3^ In May 2020, we formed the COBAP Team to document for researchers and the public a systematic database of these closures, hand-coded from government policy texts into systematized closure types (https://covidborderaccountability.org). We found more than 1000 entry restrictions introduced in 2020, marking a sudden and unprecedented halt to people’s access to travel and movement across international borders. Our primary research question is whether these policies worked to reduce the spread of the novel 2020–2021 coronavirus, SARS-CoV-2.

Our hypothesis follows the expectations from the literature of past pandemics, predicting that international border closures in response to the COVID-19 pandemic did not delay the virus’ spread unless island nations implemented border closures early in the timeline of global spread. Already, we can see from the border closures and SARS-CoV-2 data visualized on our project website that international border restrictions were not sufficient, on their own, to contain the spread of SARS-CoV-2. Timing is a key factor since, in theory, a complete border closure should bar the possibility of human-to-human transmission. For this reason, we would not expect the international border closures introduced (mostly in March 2020) to reduce spread. They were introduced after the novel coronavirus had already entered countries undetected and, in some cases, after detection. It might still be reasonable to expect that reductions on human movement via border closures could reduce further spread into the country, hence the need for this study. However, nearly every border closure introduced exceptions for essentials-related movement, its own citizens, workers, or populations of specific countries, illustrating the impracticality of border closures as well as a weakness inherent to studying them.

To test our hypothesis more concretely, we used the causal inference method of panel matching with cross-sectional data to determine, per policy category, whether the decision to implement a specific policy corresponded with a decline in reported SARS-CoV-2 spread, after controlling for underlying factors. The covariates we include are political regime dimensions (liberal democracy, freedom of expression), population demographics (logged population size, percent over 70 years old, median population age), developmental and economic factors (Human Development Index, life expectancy, logged GDP (PPP), land area), number of daily tests per capita, percentage of vaccinated population (first dose and second dose), as well as a control for the domestic lockdown policies. We, then, compare covariate balance improvement across different matching refinement strategies to select the optimal performing technique for each model. We also applied validity tests which isolated the impact of domestic lockdowns and of island countries, increasing our confidence in the results.

Neither of our non-matching, difference-in-difference models, nor any of the matching techniques yielded results in favor of international border closures. To our surprise, island countries also saw no effect. The type of border closure (complete versus partial closures or targeted entry bans based on travel history versus citizenship status) also did not impact SARS-CoV-2 spread. We found evidence in favor of domestic lockdowns, which is in line with recent pandemic studies. What is unique about our analysis is that the COBAP database and matching design allowed us to isolate the impact of international border closures. We highlight our null findings, across multiple lines of analysis as indicative against the efficacy of pandemic-related border closures as well as of the need to study their byproduct effects and compare these results to other preventative measures introduced at borders.

## Literature review: limitations and COBAP’s contributions

Studies on the impacts of international border closures from past pandemics are extremely limited. A review of the epidemiological studies on non-pharmaceutical interventions (NPIs) introduced in response to previous infectious diseases suggested that if measures were introduced early enough, virus transmission into the country was delayed only by a few days or weeks.^4^ Specifically, maritime quarantines of small islands during the 1918-19 influenza pandemic seem to have delayed the arrival of the disease. Another systematic review similarly highlighted that four islands with strict border controls during the 1918-19 pandemic reduced both the spread and mortality from influenza compared to islands that introduced no border controls.^5^ However, both of these reviews reported that the data for the studies was limited and/or of “very low” quality.

Our study addresses the data availability problem by covering decisions made by 235 country entities, including administrative units of island territories, for the timeline of available information between 1-Jan 2020 and 19-Apr 2021. This is the most comprehensive and precise record of border closures introduced in response to the pandemic, to our knowledge. Shiraef et al.^3^ reviewed comparable data sets in more depth in the initial release of the COBAP data set. For this study’s purpose, we converted the COBAP Team observational data into a time-series database of country-week units, which allows us to assess a sample size of more than 11,000 units.

Available studies on the impacts of border restrictions on the spread of SARS-CoV-2 are also limited by virtue of its novelty. This means the earliest studies implemented theoretical models instead of observational studies and/or drew inference from preliminary data limited to specific locations.^6–13^ For instance, Cowling et al.^14^ found evidence in favor of Hong Kong’s early introduction of NPIs, including border restrictions (in Jan 2020)^15^, but because the data is limited to Hong Kong, it is impossible to separate the impact of border restrictions versus the other measures introduced in the same time period (including internal movement restrictions, social distancing, and changes in population behaviour). Bou-Karroum’s systematic review of NPIs was more comprehensive in global scope and, similarly, suggested that the timing of border closures, as well as their combination with other measures, enhanced efficacy of reducing spread.^16^ However, their result related to border closures is inconclusive due to an extremely limited sample size. Shi et al. modelled the risk of SARS-CoV-2 transmission using distance between travel destinations and airline passenger data, reporting minimal impact from travel restrictions, “with almost zero (median) change in risk of virus importation.”^13^

A second limitation of previous studies on the efficacy of border closures introduced in 2020 is that most do not control for domestic responses to the pandemic, thus conflating which measures reduced SARS-CoV-2 spread. One exception to this from past pandemic data examined both international and domestic movement restrictions in response to seasonal influenza, concluding that stringent travel restrictions may delay but do not prevent local transmission of the disease.^17^ Two other exceptions to this limitation of available studies include (1) a study which investigated both international and internal travel restrictions by reviewing pre-printed and published studies by 1-Jun 2020 and (2) a subsequent 8-Jun 2020 study which estimated the effect of all non-pharmaceutical interventions but from a limited sample size of six countries with known outbreaks in early 2020 (China, South Korea, Italy, Iran, France, and the United States).^7,8^ Our study joins these two contributions by prioritizing national-level lockdowns at the global level as a validity check on our lack of findings that border closures correspond with a reduction in the spread of SARS-CoV-2. We surpass these studies by examining the combined results over a much greater length of time and after testing had become more widely available.

Third, the available studies are prone to significant bias because the outcome of interest—new SARS-CoV-2 cases—also influences the decision to initiate new policies. In other words, pandemic policy research cannot easily disentangle the policy outcomes from the policy responses. Moreover, we expect covariation with case spread and other factors, such as countries’ degrees of economic development, political regime type, demographics, and healthcare capacity, all of which reasonably influence both the spread of SARS-CoV-2, as well as our treatment (the decision to introduce new policies). This is a problem for viable causal inference, which necessitates randomized treatment assignment. To address this challenge, we leverage a statistical matching technique across comparable, relevant factors at the country-level, using the *PanelMatch* package in R.^18^ Panel matching generates a synthetic counterfactual to the treated units, allowing for a much improved causal inference strategy.

A fourth limitation of previous studies on border closures is that most implement a binary variable of whether or not a border closure was introduced on a given day or not, measured by international flight reductions. This is insufficient because countries introduced a variety of entry restrictions, with varying durations of time, and targeted at varying routes of access into a given country. Thus, the sub-categorization of the novel COBAP data set allows our study to compare a broader set of border closures, ranging from complete closures of international borders (with exceptions only for essentials, a broader category which also includes work-related visa exceptions, and/or up to 10 specific country exceptions), to partial closures (including bans targeted at specific countries based on travel history, citizenship status, or entry route—through air, land, or sea). To our knowledge, our study is the first to include sourced, verified end dates for border policies. The COBAP database allows for robust time-series analysis on health outcomes for this study as well as for a wider variety of socioeconomic outcomes for future studies.

Fifth, an inherent limitation of country-level policy analysis is that many rely on information reported at the country-level for both the independent and dependent variables. But countries vary widely on how and whether they report mortality data. They may have political incentives to under-report the spread of SARS-CoV-2 and its associated deaths following a sweeping, national-level decision like a border closure. To address this potential bias with mortality data, we recommend the Economist’s excess death model, which estimates the “true” death toll from contextual information per country.^19^ The outcome variable of this study—SARS-CoV-2 spread data—relies on the John Hopkin’s University dashboard,^20^ which draws primarily from country-reported data. However, given that the direction of our hypothesis is that international border closures do not reduce spread, this potential bias entails under-reporting versus over-stating any results. Moreover, our inclusion of health capacity as a covariate limits case reporting bias. Further, our data coverage—in both time and scope of countries whose policies are covered—is as comprehensive as available at this point in time.

## Methodology

### The COBAP data set

Launched in May 2020, the COBAP database was introduced to track systematically the new international border closures being introduced in response to the coronavirus pandemic. The data set was hand-coded by research assistants (RAs) we trained and assigned five or more countries to record new border policies each week throughout the pandemic and the dates on which they ended. RAs systematized a variable of border closures per country by filling out a 20-question survey per policy. The survey had pre-set categories of border closures that included two discrete meta-categories (“complete closures” versus “partial closures”) and eight respective, non-discrete sub-categories. A **complete closure** is when a country restricted entry to all foreigners on a given day. The sub-categories under complete closures include (1) when no exceptions are made except for essential services, such as humanitarian aid and for medical personnel (“essentials only”); (2) when exceptions are made only for citizens (“citizen exception”); (3) when exceptions are made for a broader set of work-related visas (“workers exception”); and (4) when exceptions are made for a specific set of countries, up to 10 (“specific countries exception”). A **partial closure** is when a country has restricted entry to some but not all foreigners and/or through some but not all routes of entry on a given day. The sub-categories of partial closures include: (1) when an entry restriction targets regular air, land, and/or sea routes, but not all three (“air,” “land,” or “sea” border closure) (2) when an entry restriction targets a population based on their recent travel history (travel history ban), (3) when an entry restriction targets a population based on their citizenship status (citizenship ban), (4) or when an entry restriction targets regular visa services (visa ban). RAs also completed a contextual search for each country they were assigned, contacted public officials to confirm start and end dates, and conducted a review of another RA’s set of policies recorded. The policy assignments per country are available in full in the Supplementary Information. A full overview of the COBAP data collection process is available here.^21^

Because the data set structure includes verified start dates, per day, across a large time period, it is particularly conducive to the large sample size required for finding comparable sets through matching analysis. For purposes of this analysis, we converted the data into weekly time-series data to account for potential lags in testing. We chose April 19, 2021 as the cutoff because April 20, 2021 was when the first border closure was introduced mandating vaccines for entry, which we understand as a qualitatively different type of border closure than those collected previously.

### Other public data sources used for covariates

To measure national-level policies, we relied on the Oxford COVID-19 Government Response Tracker (OxCGRT) for comprehensive national-level restrictions on movement, which we term domestic lockdowns.^22^ We restricted the *domestic lockdown* variable to only include OxCGRT variables C1:C7, removing their border-related policies (which were less precise than our database of closures between increased border screenings and actual closures). Moreover, removing their border policies allows for a more precise validity check. The seven variables that compose the lockdown index are thus: school closings, workplace closings, cancellations of public events, restrictions on gatherings, closing of public transportation, stay-at-home requirements, and restrictions on internal movement.

Additional variables included in our model are accessed from the Economist’s Excess Deaths Model data set, which was introduced to estimate the “true death toll” per country of the COVID-19 pandemic.^19^ These variables are sourced from the Varieties of Democracy (V-Dem) data set,^23^ the World Bank,^24^ and the United Nations (UN) Development Programme.^25^ We rely on SARS-CoV-2 case data from the Johns Hopkins Covid-19 Tracker, accessed December 2021.^20^

After combining the COBAP data set with these publicly available data sets, we organized panel observations into country-week units. Our first week of observations begins on January 19, 2020 and ends on February 25, 2020. This pattern continued through Apr 19, 2021, resulting in 65 weekly periods. We chose this cutoff period because it marks the last week before countries started to add vaccine requirements to border closures, which we understand as a qualitatively different type of border closure. SARS-CoV-2 case data and other covariates are not available for the first two weeks of 2020, and there are no border closure policies initiated during this time. Due to limited data for our covariate controls, our original set of 252 countries and island territories included in the COBAP data set is reduced to 185. This resulted in a maximum possible data set composed of 11,975 country-week observations.

### Outcome of interest: rate of change in new SARS-CoV-2 cases

Our outcome of interest is the rate of change in new SARS-CoV-2 cases, controlling for population size per country. This provides a more accurate outcome of interest than raw case counts. If a policy effectively reduces disease spread, the rate should be lower in subsequent weeks regardless of whether the rate of new cases was already rising or falling at the time of the treatment. Our data for this variable was sourced from the COVID-19 Data Repository by the Center for Systems Science and Engineering (CSSE) at Johns Hopkins University.^20^

First, cumulative new daily case counts were summarized into weekly values by taking the end-of-week value. The new cases per week are then transformed into per capita figures for each 100,000 persons using each country’s population size. Next, we subtract this value in $\hbox {T}_{-1}$ from $\hbox {T}_{0}$ to produce the rate of change of new weekly cases per 100,000 country residents. Finally, in order to neutralize influential outliers and improve normality, we conduct an Inverse Hyperbolic Sine Transformation (IHST) to reduce kurtosis. The large differences in the new cases week to week appear to be primarily by countries with small populations. Positive values represent an increase in the number of new SAR-CoV-2 cases per capita, whereas a negative value signals a decline in new cases from the previous week. See the Supplementary Information for further discussion of this variable’s distribution and transformation.

The week of treatment is specified as $\hbox {T}_{0}$. We select a “lag” of three units, meaning that the control time periods used are $\hbox {T}_{-1}$:$\hbox {T}_{-3}$. Selecting the lag value requires a balance between capturing relevant information in the pre-treatment period and limiting the period in order to not censor data unnecessarily. The resulting matched sets are composed of control observations that have precisely the same treatment history during the lag period as the treated observation. We select a “lead” of five units, meaning that quantities of interest are calculated for $\hbox {T}_{0}$:$\hbox {T}_{5}$, or the week of treatment to five weeks after. Estimates from available literature^26,27^ observe a 5–22 day incubation period for SARS-CoV-2. Thus, we expect that the impact of effective policy interventions should be apparent by $\hbox {T}_{+2}$. We also focus on $\hbox {T}_{+2}$ because testing data may lag up to a week from point of infection. Moreover, $\hbox {T}_{+2}$ is when we observe the strongest reduction in spread following domestic lockdowns. For maximum transparency, we report the results of SARS-CoV-2 spread for all weeks $\hbox {T}_{-3}$:$\hbox {T}_{5}$.

### Global trends of new border policies introduced in response to the COVID-19 pandemic

**Figure 1:**
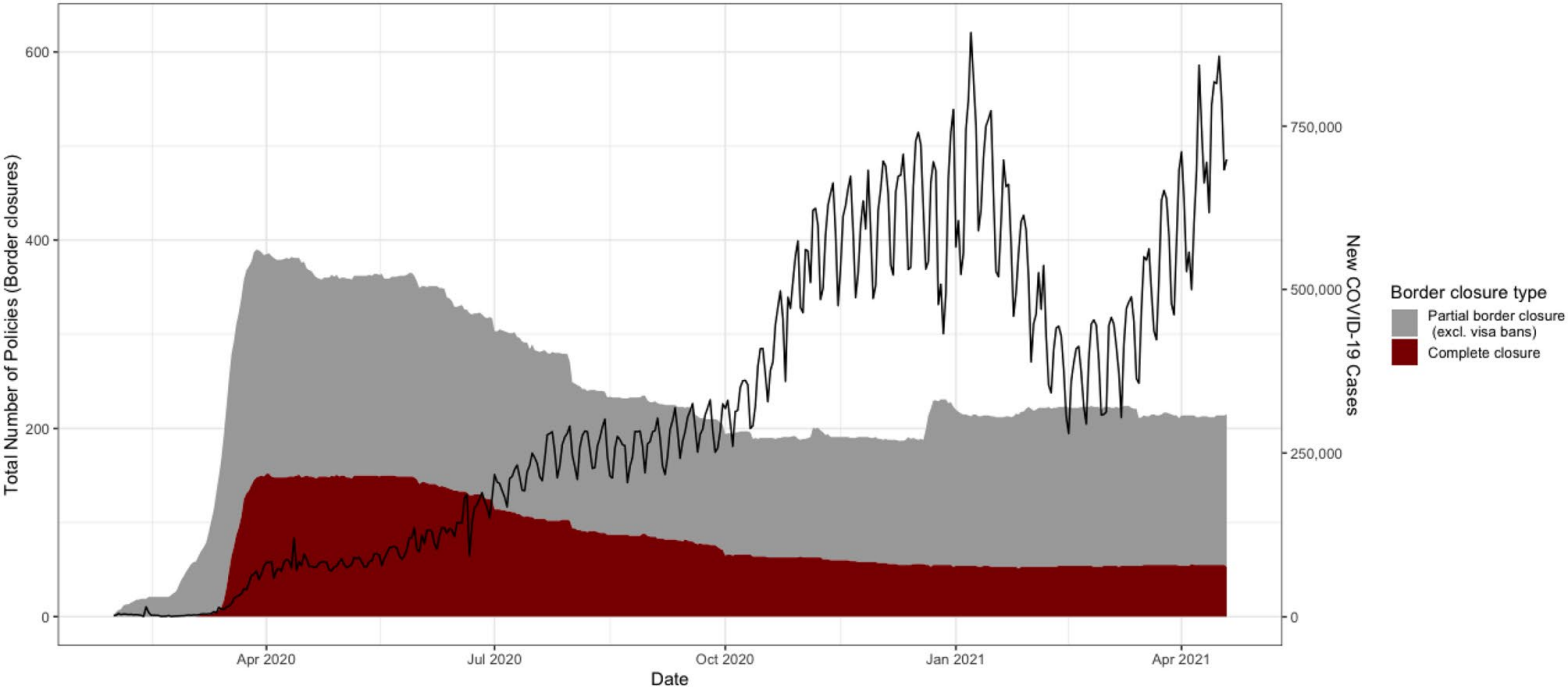
Descriptive data of border closure policies and new Covid-19 cases from January, 2020 until April, 2021.

In this section, we describe the trends of complete and partial border closures on a global level. Figure 1 shows the total number of complete and partial closures and the growth in reported new SARS-CoV-2 cases^19^ from January 31, 2020, until April 19, 2021. Following a rapid increase in the number of complete closures in March—and an ensuing peak of 154 policies enacted—we observe that more than 130 complete closures were in place between April and June. From June until the end of the year, the number of complete closures declined non-linearly until reaching a minimum of 50 in December 2020. The number of partial border closure policies implemented also rose sharply in March. Yet, the number of partial closures introduced decreased at a slower pace throughout the year. In December, there was a notable increase in the number of partial closures. From January until April 2021, the number of partial closures decreased slightly. Figure 1 also illustrates a growth in reported SARS-CoV-2 cases on a global level. The data reflects steady growth in reported global cases throughout 2020, followed by a sharp decline in late December—before returning to previous levels. We observed a downward trend in global daily new infections from January until March.

Due to variation in the testing and healthcare infrastructures of countries, the raw case data does not allow a causal claim about aggregated complete and partial border closures on the global outcome of case spread. To address this insufficiency in the data, we employ a panel matching method—detailed below—which allows more precise comparisons between countries with similar underlying factors interacting with the rate of SARS-CoV-2 spread in a given country.

## Matching analysis

This section assesses whether border closures were effective in reducing the spread of SARS-CoV-2 cases using matching analysis. We designated from the COBAP database the onset of a new border closure introduced during our period of analysis as the treatment group. Because these border closures were not randomly assigned, we generated a more accurate causal estimate through a matching technique across comparable country-level factors.^28,29^ Matching involves a systematic selection of similar cases to approximate a counterfactual for each treated case. In other words, our analysis compares countries with a new border closure policy (treatment) to similar countries that did not institute the policy (controls). Since panel data varies over time, matching allows control units to include both the time leading up to the “treatment” for a given country of interest as well as the same period for similar observations that were not treated.

We estimate the effects of different border closure policies included in the COBAP data set using the *PanelMatch* package in R.^30^ The policy effects are quantified through a nonparametric generalization of difference-in-differences, with $\hbox {T}_{-1}$ serving as the baseline. In simulations, the package authors show that panel matching estimation reduces bias compared to the standard two-way fixed effects regression technique commonly used in panel studies.^31^ In order to find the most comparable control conditions, the *PanelMatch* package is useful because it both matches and refines the data to generate matched sets for the relevant treatment units.

First, we specify the covariates or controls on which refinement occurs. These included the political, economic, healthcare, testing, and domestic policies specified above, as well as relevant COBAP policy variables. For the country-year covariates, we do not select a time period as they do not vary across weeks, while for our time-varying covariates, we specify selection for the entire period of analysis ($\hbox {T}_{-3}$:$\hbox {T}_{+5}$). We then calculate and compare different refinement strategies to assess which is best able to minimize covariate imbalance during the per-analysis period ($\hbox {T}_{-3}$:$\hbox {T}_{0}$). The balancing assessment results can be found in the Supplementary Information for our three treatment variables.

Once matched sets are designated for the select treatment observations and refinement is conducted on the covariates to increase balance, the quantity of interest—the average treatment effect for treated units (ATT)—can be calculated. This is accomplished by taking the difference between the control sets for each treated unit and generating a weighted average for each of the lead time periods. Finally, standard errors are computed using 1,000 weighted bootstrap samples.

### Domestic lockdown validity check

We first conducted a validity check on our model design. We test the effects of a significant increase in domestic policy restrictions as represented by the lockdown variable. Because the index is a continuous variable, we select weekly changes that represent a significant increase in strictness of 0.25 or more to create the *domestic lockdown* treatment variable. This occurs in 253 (2.1%) country-week observations.

**Figure 2:**
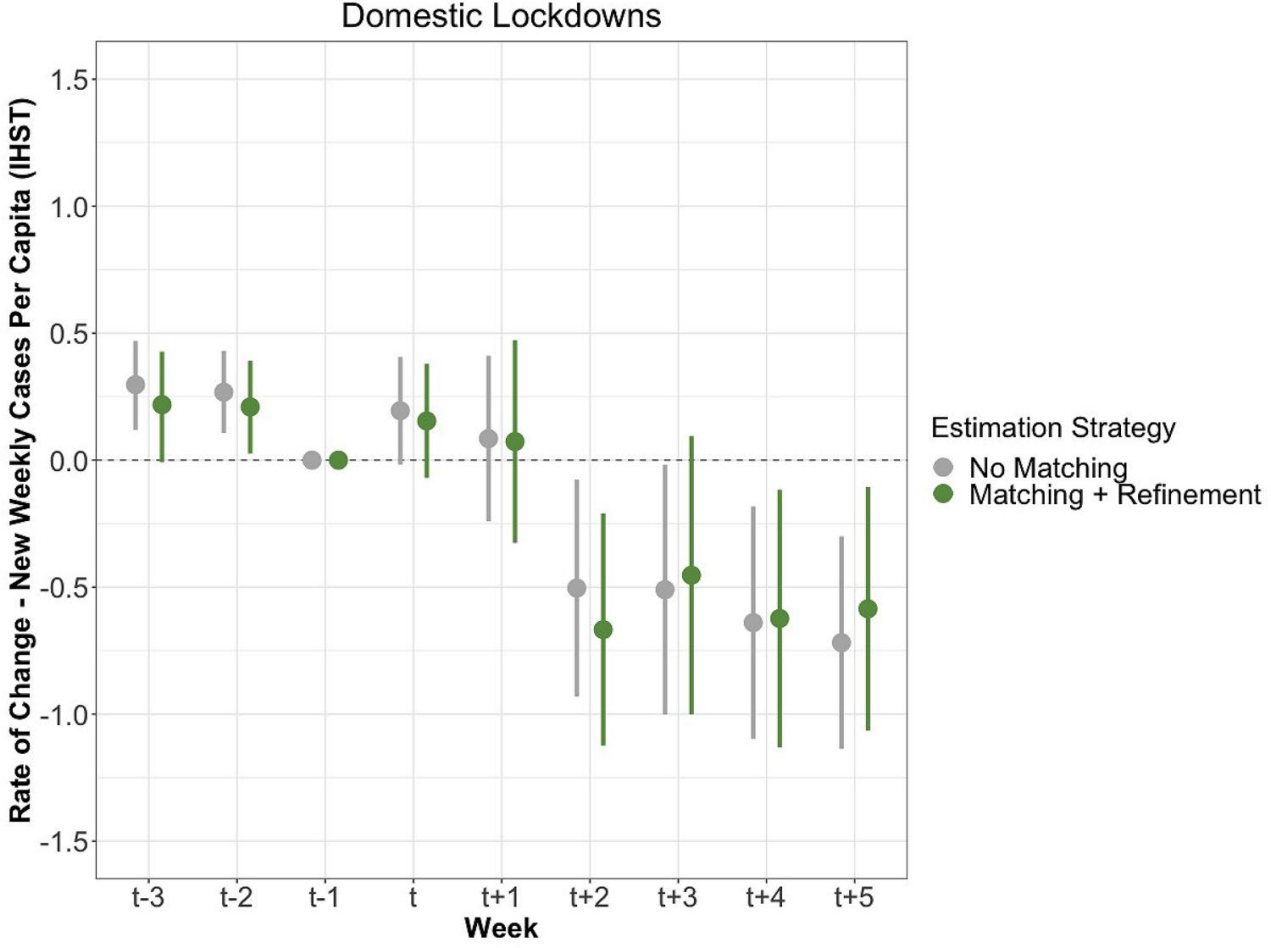
This figure illustrates the measured effects of a domestic lockdown on the rate of change of new cases per capita after IHST (y-axis). The estimates in grey were generated with neither matching nor refinement, while the green estimates were generated with matching and refinement, both displayed with 95% confidence intervals. The refinement strategy selected is covariate balancing propensity score matching. The period includes nine weeks, three prior to the lockdown, the week of the lockdown, and five following the lockdown.

For a policy to be effective, we anticipated a decline in new cases per capita, especially at around $\hbox {T}_{+2}$. Figure 2 presents the estimated effects of the lockdown treatment. First, we note that case rates are increasing at a statistically significant rate in our pre-treatment periods $\hbox {T}_{-3}$ and $\hbox {T}_{-2}$. The rate of new cases is still increasing in both $\hbox {T}_{0}$ and one week later in $\hbox {T}_{+1}$, but at a more moderate pace. At $\hbox {T}_{+2}$, however, new cases per capita decrease at a statistically significant rate compared to the baseline of $\hbox {T}_{-1}$. The upper $\hbox {T}_{+2}$ confidence interval is well below the baseline estimators $\hbox {T}_{0}$ furthermore, for weeks $\hbox {T}_{+3}$:$\hbox {T}_{+5}$, the rate of new SARS-CoV-2 cases diagnosed continued to fall. We interpret this finding as lockdowns demonstrating a strong, negative, and persistent effect on virus spread.

### Complete and partial border closures

Next, we test the effectiveness of border closure policies categorized as *complete closures* or *partial closures*, according to the COBAP coding scheme. The matching process yields 204 matched sets out of 215 treatment observations for complete closures, and 535 sets out of 759 treatment observations for partial closures. Aside from the treatment variable assignment, all other model parameters remain the same as was the case for the lockdown model above.

**Figure 3:**
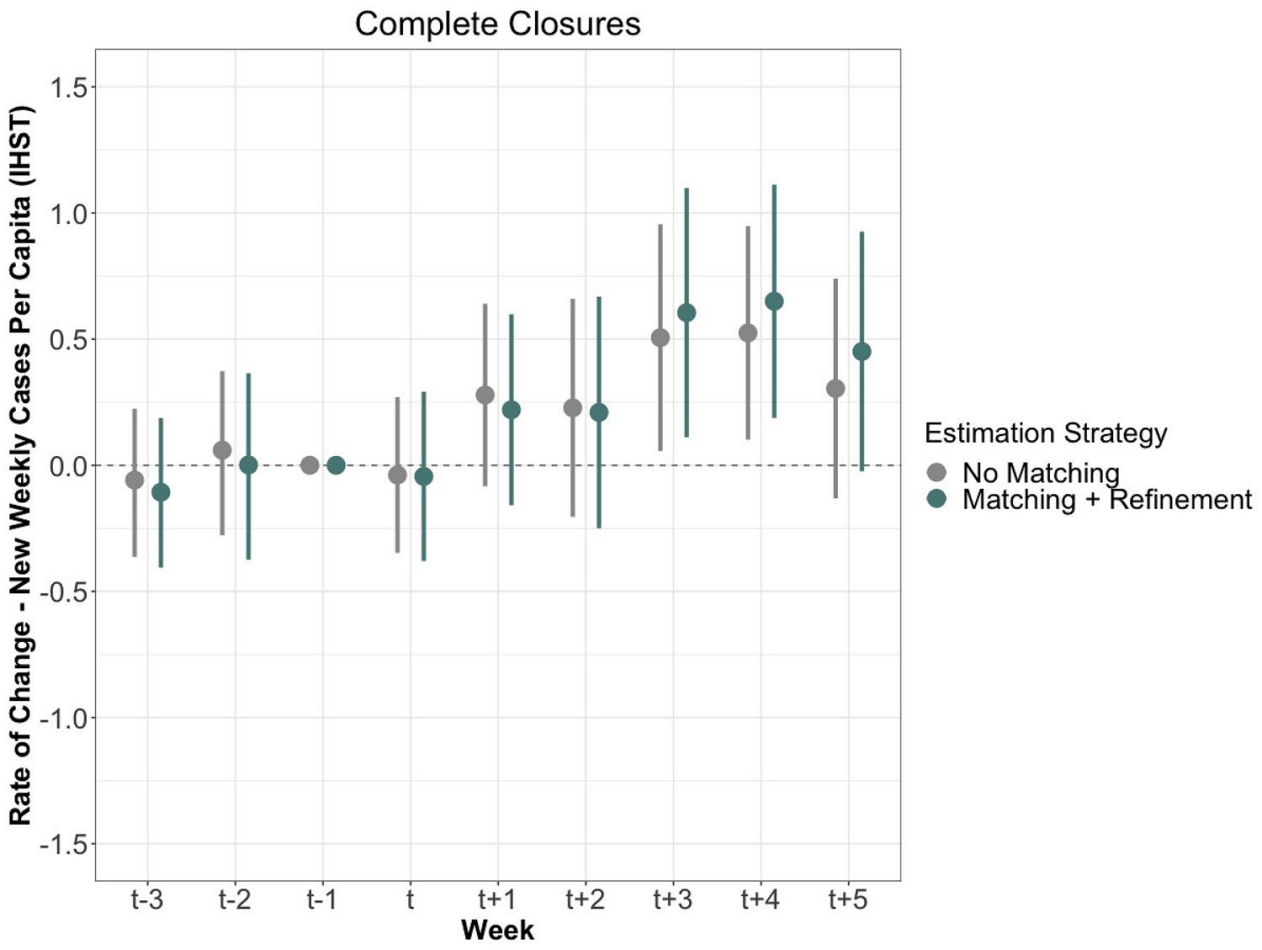
This figure shows the measured effects of complete closures on the rate of change of new cases per capita after IHST (y-axis). The estimates in grey were generated with neither matching nor refinement, while the blue estimates were generated with matching and refinement, both displayed with 95% confidence intervals. The optimal refinement strategy selected is propensity score matching. The period includes nine weeks: three prior to the border closure, the week of the closure, and five following the closure.

**Figure 4:**
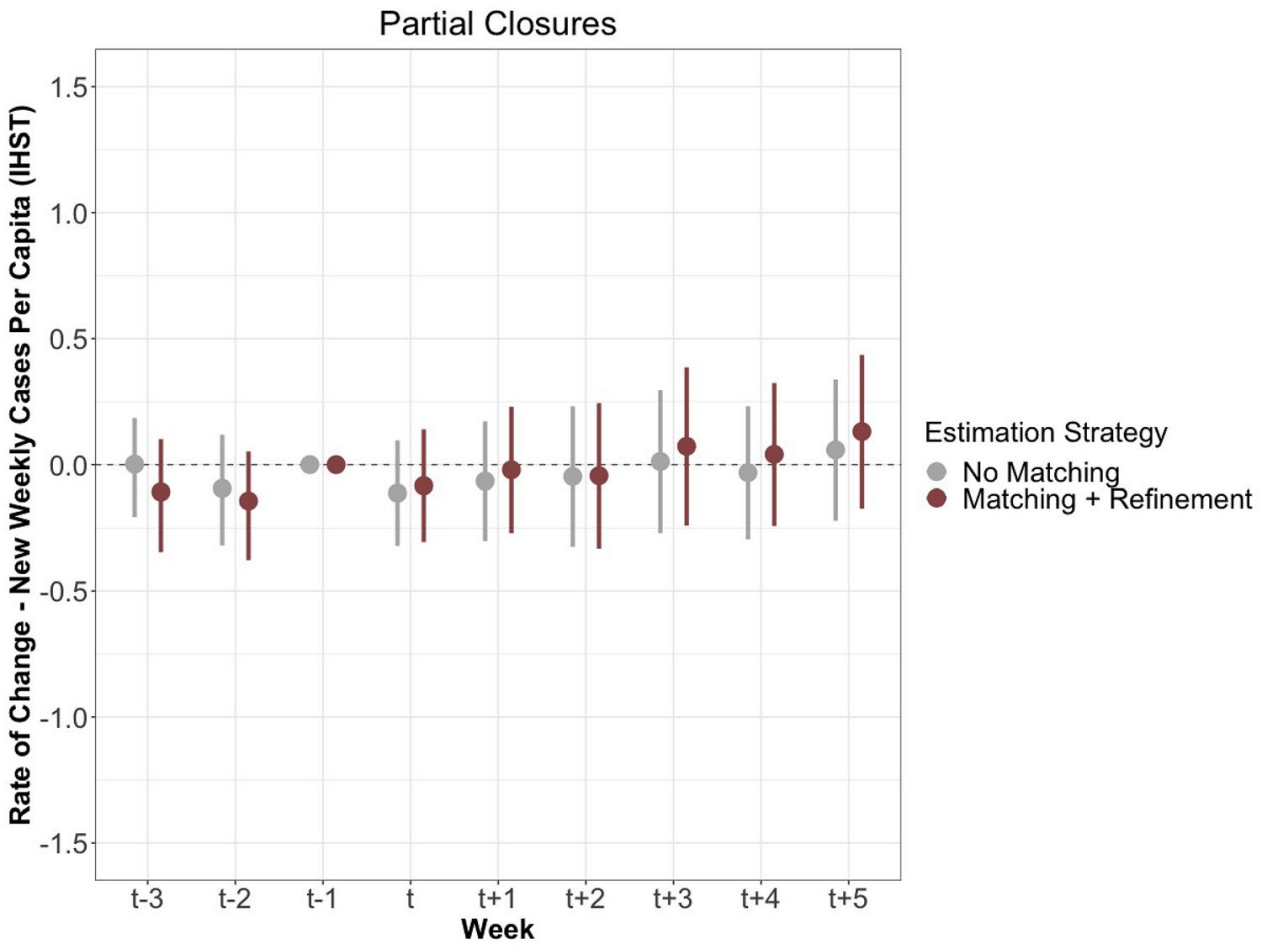
This figure shows the measured effects of partial closures on the rate of change of new cases per capita after IHST (y-axis). The estimates in grey were generated with neither matching nor refinement, while the red estimates were generated with matching and refinement, both displayed with 95% confidence intervals. The optimal refinement strategy selected is covariate balancing propensity score matching. The period includes nine weeks: three prior to the border closure, the week of the closure, and five following the closure.

Figure 3 presents the estimated effects of complete border closures for the three weeks prior to and five weeks following the start of a new policy. The main effects of interest are shown in blue, while non-matching estimates are shown in grey for reference. In contrast to domestic lockdowns, the complete border closures were not effective at decreasing SARS-CoV-2 spread. The $\hbox {T}_{+2}$ estimates show that rates increase compared to the $\hbox {T}_{-1}$ baseline. In general, we observed a steady and significant increase in virus spread across the entire time period. Weeks $\hbox {T}_{+3}$:$\hbox {T}_{+5}$ further show that cases increased at a statistically significant rate from the baseline measure. We interpret this rise as indicative of an ineffectiveness of border closures to halt the rapid spread of cases around the globe.

Most complete closures were implemented in March 2020, when reported case count of SARS-CoV-2 was low, relative to the rest of the year. As such, if border closures effectively reduced spread, and early timing was a significant factor, we would most expect a reduction of cases in this period. Instead, case rates accelerated thereafter, indicating a lack of efficacy for the category of complete closures which made exceptions for citizens. The increase could also be explained by the added incentive of this policy type to travel, with citizens rushing home in this period and/or non-citizens suddenly deciding to exit.

We also analyze the effects by sub-type of complete closure as shown in Table 1. While none are statistically significant from the baseline, the essentials-only complete closures generated a negative estimate at $\hbox {T}_{+2}$. This is in line with the theoretical expectation that the strictest border closures, if reducing all movement across the border, should reduce viral transmission of the virus. However, the character of this policy also meant that it was rare, resulting in the smallest sample size in our data set.

The results from a second set of policies categorized as partial border closures are presented in Fig. 4. Again, the results highlight the ineffectiveness of partial closures at decreasing SARS-CoV-2 spread. The estimate at $\hbox {T}_{+2}$ is extremely close to $\hbox {T}_{-1}$, and at no point are estimates statistically different from this baseline. Given the greater data availability and spread across time, the partial closures provide a more comprehensive test of border closures and strongly suggest an overall null effect in relation to a reduction of new cases.

### Summary of policy outcomes by sub-type

Table 1 reports the effects and models across the different border closure types, alongside the total number of available treatment units, the number of matched sets, optimal refinement strategy, the difference between no refinement and optimal refinement covariate imbalance, and the $\hbox {T}_{+2}$ estimates and confidence intervals, the key week we expect to observe a decline in the rate of new cases. The effect size for domestic lockdowns is -0.667, and the difference between $\hbox {T}_{-1}$ and $\hbox {T}_{+2}$ is statistically significant at the p<0.05 level. The change in the rate of new cases for all complete border closures is positive but not statistically significant, while the estimate for all partial closures is negative and not statistically significant.

Next, we conduct panel matching models for all eight of the COBAP policy sub-types, the first four of which were categorized (prior to data collection) as “complete closures” and the latter four as “partial closures.” These results, presented by sub-type, are less robust due to the smaller sample sizes, and none are statistically significant at the $p< 0.05$ level. Complete closures that exempted citizens were highly concentrated in late March 2020 as preventative measures. Most of these policies (60%) were implemented during the two weeks from March 15 to March 28. Between March 15 and April 15, the global total of daily new cases rose steeply as reported cases of the virus increased. Rather than indicating that border closures increased SARS-CoV-2 spread, this correlation suggests that more research into the specific types of closures and internal measures is needed.

The umbrella category of partial border closures offer greater statistical power for analysis than the complete closures because they are best represented in our data set. For each sub-type of partial closure, we find null results. We interpret this as indicative against the efficacy of partial closures, whether introduced based on objective, indiscriminate criteria (such as recent travel history) or discriminate criteria (such as citizenship status). These are preliminary results, however, and causal claims about specific types of border closures are beyond the scope of this study. We report them for transparency purposes and to invite further research on the topic.

**Table 1:** Summary of model efficiency and results by general and specific policies.

Policy intervention	Treatment	Matched	Refinement	Pre-refine	Post-refine	$\hbox {T}_{+2}$	$\hbox {T}_{+2}$
	Units	Sets	Method	Imbalance	Imbalance	Estimate	(95% CIs)
OxGRT domestic lockdowns	253	191	CBPS	0.088	0.050	$-$ 0.667	$-$ 1.106:$-$ 0.216
**COBAP complete closures**	215	204	PS	0.128	0.034	0.210	$-$ 0.248:0.698
Specific country	20	18	PS	0.556	0.039	0.922	$-$ 1.397:3.235
Work exception	69	59	CBPS	0.294	0.010	$-$ 0.095	$-$ 0.889:0.614
Citizen exception	106	103	PS	0.059	0.029	0.336	$-$ 0.100:0.758
Essentials only	24	23	CBPS	0.109	0.028	$-$ 0.453	$-$ 1.129:0.209
Islands (subset)	47	47	Mahal.	0.128	0.107	0.727	$-$ 0.245:1.884
**COBAP partial closures**	759	535	CBPS	0.246	0.009	$-$ 0.044	$-$ 0.341:0.232
Visa ban	55	48	CBPS	0.072	0.001	0.288	$-$ 0.280:0.877
Citizenship ban	106	78	PS	0.368	0.029	$-$ 0.458	$-$ 1.273:0.271
Travel history ban	131	84	PS	0.244	0.013	$-$ 0.445	$-$ 1.151:0.201
Border closures	518	381	PS	0.127	0.002	0.026	$-$ 0.304:0.372
Islands (subset)	143	87	PS	0.364	0.289	0.029	$-$ 0.634:0.558

The first two columns show the total number of treated units available for analysis and then the number remaining after matching. Columns three through five report the refinement method used for each model as well as the reduction of imbalance between a model without refinement and after refinement, with 0 representing optimal balance across all variables. Finally, column six reports the $\hbox {T}_{+2}$ estimate, and column seven provides the 95% confidence intervals.

It is noteworthy that the pre-treatment periods for domestic lockdowns are positive and statistically significant, while the estimates for border closures are negative, though not statistically significant. This suggests a different rationale for instituting lockdowns compared to border closure policies. For domestic lockdowns, SARS-CoV-2 cases were, on average, increasing at a significant rate, which implies a clear justification for the implementation of restrictive policies. This raises a question on the motivations behind border closure policies, if not meaningful rises in new SARS-CoV-2 cases. Different types of restrictive policies occurred at different time periods, which likely influenced the results even after the matching process. As such, these results are preliminary and should be interpreted in relation to domestic lockdowns cautiously.

### Islands test

**Figure 5:**
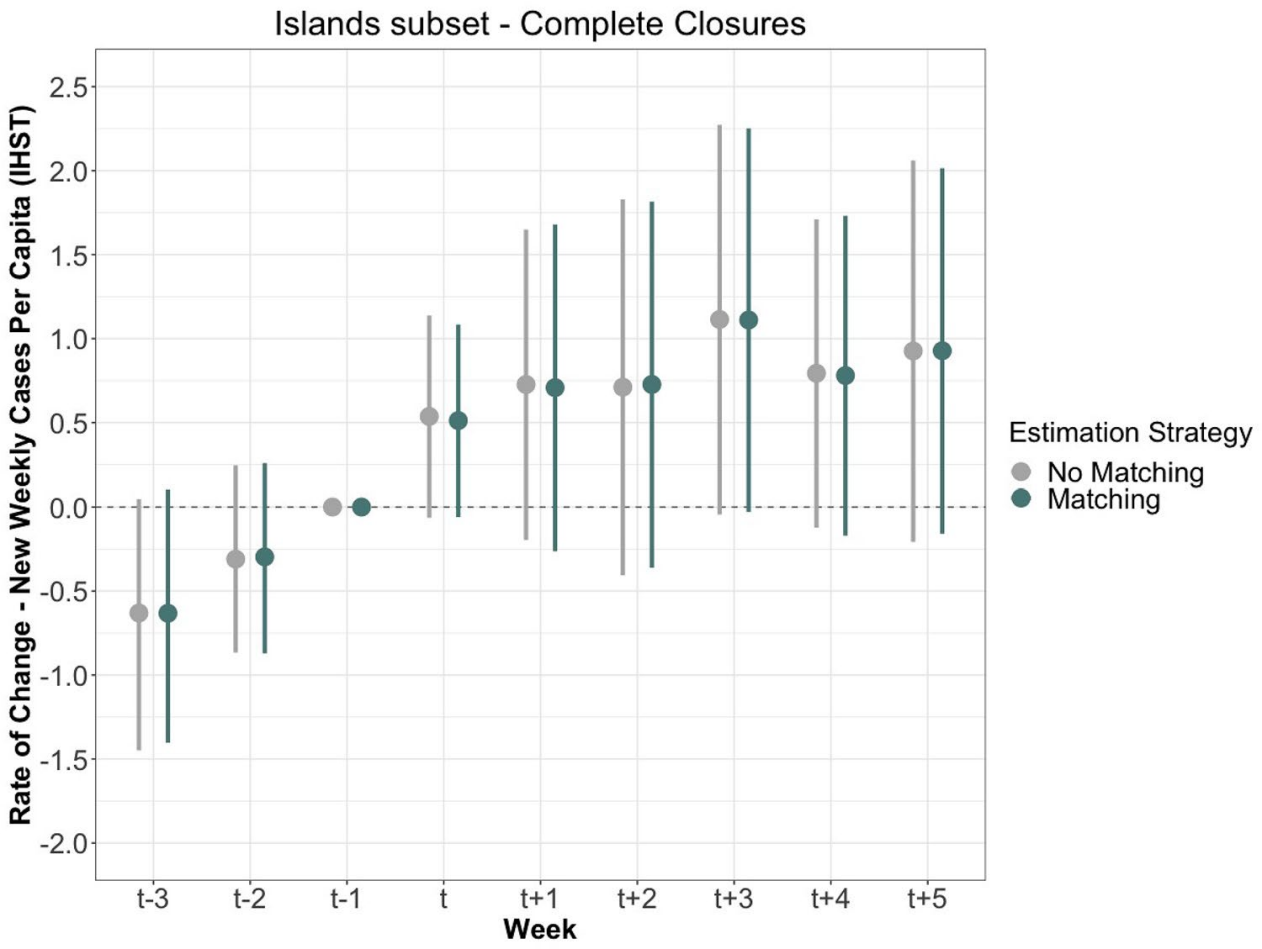
The measured effects of complete closure on the rate of change of new cases per capita after IHST (y-axis) for a sub-set of 89 island nations. The estimates are shown with both no matching or refinement in grey, and matching and refinement in blue, calculated with 95% confidence intervals. The period includes nine weeks: three prior to the lockdown, the week of the lockdown, and five following the lockdown.

**Figure 6:**
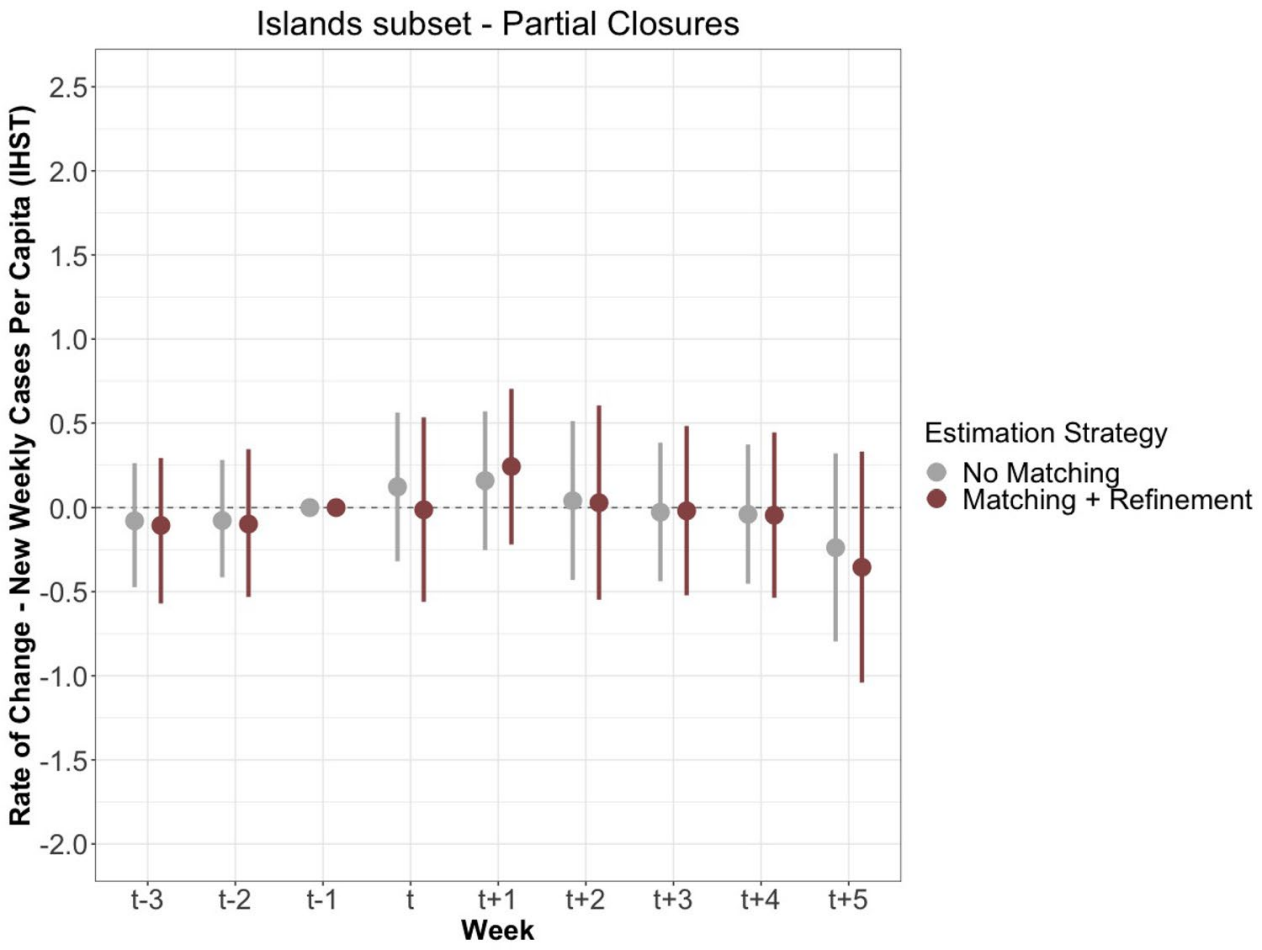
The measured effects of partial closure on the rate of change of new cases per capita after IHST (y-axis) for a sub-set of 89 island nations. The estimates are shown with both no matching or refinement in grey, and matching and refinement in red, calculated with 95% confidence intervals. The period includes nine weeks: three prior to the lockdown, the week of the lockdown, and five following the lockdown.

We further assess the effectiveness of complete and partial border closure policies by restricting data to only island countries. Literature on past pandemic, in line with WHO advice, indicates that border closures may be more effective for small island countries. The matching process yields 47 matched pairs out of 47 treatment observations for complete closures, and 99 matched pairs for 143 treatment observations for partial closures. Figure 5 displays the results for complete closures, and Fig. 6 shows the results for partial closures.

The findings closely mirror the overall sample results. For complete closures, the rate of new cases per capita standardized (IHST) increased steadily starting from the pre-treatment period. Again, we find that the rate of change is higher in $\hbox {T}_{+2}$ than in $\hbox {T}_{-1}$ baseline. Unlike the main sample finding, however, none of the post-treatment estimates are statistically significantly different from the baseline at the p<0.05 level.

Partial closures for island countries, shown in Fig. 6, show little difference in virus spread following treatment, with the $\hbox {T}_{+2}$ estimate almost exactly at the same level as the baseline. While these sub-set samples experience larger standard errors, reducing our confidence in making a clear assessment, we generally find no significant decrease between the rate of new cases and complete and partial closures for island countries. See Supplementary Information for the effects of the lockdown index on new SARS-CoV-2 cases, restricted to island countries.

## Discussion

This research agenda emerged from the authors’ uncertainty about the impacts of international border closures introduced in response to the COVID-19 pandemic.^32,33^ On the one hand, the WHO had advised against international border closures in response to the pandemic. On the other hand, the novel nature of the virus resulted in many unknowns around transmission routes and effects in early 2020. Forecasting studies did not rule out the need for border closures in the earliest efforts to contain the first known strains of SARS-CoV-2.^34^ As such, the first critical step of this study was to record systematically all new policies introduced at international borders. Given the crisis nature of the data, we made the data available to the public prior to publication through our project website (in Aug 2020), an online repository (in Dec 2020),^35^ and the media (in Mar 2021).^36^ We also published descriptions of the border closures data for contexts which traditionally see high rates of international travel, such as the United States,^37^ the European Union,^38,39^ Russia,^40^ and Greater China.^15^

Given the uncertainties of whether border closures were reducing virus spread, we took a data-driven approach and committed in advance to running multiple lines of analysis and publishing the results regardless of the study’s outcome. The dataset structure and pre-set criteria for inclusion did not change throughout the data collection process. Our data-driven hypothesis was that international border closures were not effective at reducing SARS-CoV-2 spread. Our results are discussed below using Stanford University’s Immigration Policy Lab (IPL) Null Results Guidelines.^41^

### Null results: border closures

In line with our hypothesis, we observe a null result for the impact of border closures. We interpret this finding as worth publishing because of the study’s statistical power, careful measurement strategy, research design, and policy relevancy. Its two central weaknesses relate to potential spillover effects between treated and non-treated units and the theoretical possibility that border closures have varying results on virus spread based on factors not included in our analysis.

#### Statistical power of the study

Our sample size (>11,000 units) is sufficient to expect an effect, in line with or surpassing the sample sizes of other studies on this topic. It should be noted, however, that the power of our panel match analyses are contingent on the number of matched treatment observations, reported in table 1 above, per sub-type. This means inferences drawn about the effectiveness of border closure sub-types are limited by large standard errors. However, the overall statistical power, for all border closures in our sample, is significantly higher than in previous studies on this topic—sufficient to trust the null result.

#### Measurement strategy

We are confident in our measurement strategy of treated and control units because their assignment was pre-defined by our project database’s criteria for inclusion, available in the initial publication of the data set.^3^ The outcome variable—a reduction in SARS-CoV-2 cases—relies on the data collected by the Center for Systems Science and Engineering (CSSE) at Johns Hopkins University (JHU).^20^ To account for the fact that countries had uneven access to testing, we included testing in our model and measured the outcome as a proportion relative to population size. Moreover, we cover a large enough time period (65 weeks) to include data after testing became available in most countries of the world. Still, we note that there is an inherent weakness of potential bias in a measurement strategy that gathers variables from government sources. To account for potential under-reporting, we included regime-type as a covariate in the model.

#### Implementation of experimental design

The matching technique in our study is a common strategy to improve causal inference of observational data. We chose to implement matching for the practical reason that our outcome variable cannot be replicated in a lab environment, and thus, is measured in a non-experimental or “real-world” setting. Moreover, our study’s treatment assignment, the decision to implement a border closure, is likely related to underlying factors of each country. While this is a relatively new modeling approach for panel data, we increased our confidence in the null result after receiving reviews from experienced statisticians.

#### Spillover or contamination of control and treated groups

A weakness inherent to our study is a potential for spillover between the treated and control groups. Because of the nature of the treatment assignment—border closures—spillover from the treated group into the control group is plausible when certain countries opted in the same week not to introduce border closures because their neighboring countries did. However, in our data piloting stage, we noted this occurring in the data in such limited cases that we opted to record only decisions made by national governments. More often, we noted a “bandwagon effect” of countries copying each other’s border closures in similar time periods. We also noticed in limited cases, retaliation policies being introduced which targeted the populations of countries that had introduced bans against theirs. Some countries may have chosen to not introduce border closures because most travellers access the country through other routes that were already impacted by a border closure, which our collected data may not account for.

Spillover in the other direction—from the control to the treatment group—is less likely because we only recorded border closures with strong sources and ones we understand were enforced on a given day.

#### Theoretical issues

Given the novelty of the virus and our initial uncertainty about the impacts of international border closures on virus spread, there may be other omissions from our model relevant to the virus which have escaped our knowledge. For instance, the advent of access to vaccines presented a theoretical challenge to our initial model (as conceptualized in October 2020). In response, we added vaccines to the final model (which did not alter the results). We have made every attempt to include relevant covariates using the best available data. There were no country-level covariates we wanted to include but on which we were unable to find information.

Another theoretical issue is that border closures may interact with virus spread differently in different contexts. Except in island countries—which we account for above—we expect this to be unlikely. Claims related to different variants of SARS-CoV-2 are beyond the scope of this study.

A central issue in our study design is that the outcome variable of interest—new SARS-CoV-2 cases—is related theoretically to the decision to introduce a restrictive policy in the first place. This is a weakness faced by most policy research since policies tend to be introduced in response to a problem already existing outside of an experimental lab. We address this problem in our study with the matching and refinement strategy which improves our control-treatment group comparison when treatment assignment is non-random.

## Overall conclusions

We found no evidence that the international border closures recorded in the COBAP database contributed to a reduction in SARS-CoV-2 spread. We found, rather, that domestic lockdowns corresponded with a decrease in new cases. Without more data, little inference can be drawn, but we believe these null results are worth reporting given the widespread and long-term impact of border closures on millions of people. This is line with recent studies that have highlighted their negative socioeconomic impacts, such as their threat to border integration in Europe, revival of territorial conflicts, xenophobia, and the often enduring negative emotional experiences of those who encounter them.^42–44^

Our most surprising finding was that island countries and territories which introduced complete closures did not then see a drop in SARS-CoV-2 spread. We recognize that our panel matching estimation strategy still suffers from bias, particularly given the significant concentration of new border closures in time and an occasional overlap of different kinds of partial closures within the same time period. In spite of these limitations, we believe this estimation strategy provides the most robust assessment of the effects of border closure policies on SARS-CoV-2 spread to date.

Our overall results indicate that domestic-level policies introduced to curb human movement within a country were more effective in response to the coronavirus pandemic than closing international borders.

Our sub-type results indicate complete closures could have had a reverse effect, contributing to SARS-CoV-2 spread. Our restricted island analysis indicates SARS-CoV-2 entered several island nations before their border closure was introduced. Without justification that international border closures reduced virus spread, future research on these border policies should ask which factors spurred them and why so many are still in place. At the time of writing (Dec 20, 2021),>250 international border closures related to the COVID-19 pandemic are in effect. Several border closures were recently introduced in response to the scientific discoveries of the omicron variant of SARS-CoV-2.

Moreover, since policymakers are tasked during a pandemic with weighing potential health benefits against the socioeconomic costs of closures, additional studies are needed to account for the human costs of these sweeping border closures. We hope others are able to build upon our results to further assess the cost, benefits, and feasibility of various domestic and international policy measures.

## Supplementary Information

Supplementary Information.

## Data Availability

All data and code required to reproduce the results presented in the manuscript are available on *Harvard Dataverse*.^45^ For access to the entire COBAP data set, researchers can use our data descriptor.^3^ Members of the public and policymakers can access the data and sources by clicking on a country in the interactive map on our project website (https://covidborderaccountability.org).
