# Supplementary material for: Did border closures slow SARS-CoV-2?
Source: Sci Rep. 2022 Feb 1;12:1709. doi: 10.1038/s41598-022-05482-7 (PMC8807811; doi:10.1038/s41598-022-05482-7)
Supplement: Supplementary file 1 — Supplementary Information. [file 41598_2022_5482_MOESM1_ESM.pdf]

Did Border Closures Slow SARS-CoV-2?

Supplementary Document: The Names Behind the Data

covidborderaccountability.org

Questions/concerns? Contact the PI responsible for the COBAP data, Mary A. Shiraef, mshiraef@nd.edu.

Contents

1 Project Overview **2**

2 RA Contributions to the Data Collection Process **3**

3 RA Contributions to the Data Review Process **4**

4 Islands Only: Effects of Domestic Lockdowns **5**

5 Construction and Analysis of Dependent Variable **6**

6 Covariate Balancing Analysis **10**

7 Average Balancing by Policy and Refinement Strategy **14**

1 Project Overview

The COVID-19 pandemic spurred wide-spread policy changes at the national level, including a broad set of border closures. In response, the COVID Border Accountability Project (COBAP) Team curated a systematized dataset of new country-level restrictions on movement across international borders.

The weekly data collection process was carried out by trained research assistants (RAs) responsible for finding and recording all the policies introduced by at least five countries during the 2020 year. RAs were aided in their process with (1) a list of evolving project resources to review each week for new policies; (2) an RA Manager, who identifies and assigns potential policies to be reviewed for inclusion into the COBAP dataset; and (3) a curated 20 question Qualtrics survey, with which RAs located the ideal source text, archived it on the Wayback Machine, read the text, and categorized the policy type according to pre-set policy definitions. A full description of the COBAP dataset—and open access—was published in the Nature Portfolio's *Scientific Data*. It can be accessed here.

Data collection is an arduous task, often requiring second language expertise and additional research on a given country's border context. This document serves to recognize specifically which RAs are responsible for the data collected per country, listed in section 2 below. In addition to data collection, each RA was responsible to complete a review process which involved contacting public officials for each of their assigned countries as well as reviewing another RA's portion of the dataset. Recognition for the data review process per country is detailed in section 3. Sections 4-7 report the results of additional analyses we ran, restricting on island countries (4), determining the measurement strategy for the dependent variable (5), balancing the covariates used in our model (6), and implementing refinement to the model for the sharpest possible comparisons (7).

As part of our commitment to accountability and open data behind the project, we commit in advance to fixing any issues raised against a specific policy the database in the three month period after publication. If you are interested in hiring a COBAP Team Member, you can contact them individually through their LinkedIn accounts available on our project website's team page.

2 RA Contributions to the Data Collection Process

Hawraa Al Janabi was responsible for the policies of the following countries: Turkey, Greenland, American Samoa, Cocos/Keeling, Faroe Islands, Martinique, Trinidad and Tobago, Tuvalu, Samoa, Tokelau, South Korea, Uzbekistan, Belize, Saudi Arabia, Jordan, UAE, Oman, Qatar, Syria, Nepal, Iran, Lebanon and Palestine.

Elizabeth Beling covered the policies of the following countries: Falkland Islands, Pitcairn (Islands), Isle of Man, Jersey, Montserrat, Turks and Caicos Islands, Virgin Islands (British), Sint Maarten (Dutch part), Northern Mariana Islands, Antarctica, Andorra, Bouvet Islands, Puerto Rico, Guam, Svalbard and Jan Mayen, U.S. Virgin Islands, Aland Islands, Bonaire, Federated States of Micronesia, Guyana, Libya, New Zealand, Tanzania.

Jonathan Falcone was responsible for the policies of the following countries: Iceland, Turkmenistan, Canada, Australia, Egypt, Nigeria, Sierra Leone, Algeria, Croatia.

Lukas Feddern was responsible for the policies of the following countries: EU/Schengen countries, Germany, Austria, Liechtenstein, Switzerland, Luxembourg, the Netherlands, Belgium, Lithuania, Finland, Denmark, Estonia, San Marino, Monaco, Italy, Ireland.

Cora Hirst covered the United Kingdom, Dominica, Haiti, Saint Lucia, New Caledonia, Thailand, Lao, Cambodia, Indonesia, Bangladesh, Bhutan, Mongolia, Rwanda, Niger, Mayotte, and Swaziland.

Nora Murphy was repsonsible for the policies of the following countries: Guadaloupe, Democratic Republic of the Congo, Congo, Barbados, Cook Islands, Saint Martin (French part), Croatia, Sweden, Latvia, Armenia, Azerbaijan, Spain, Argentina, Uruguay, Colombia, Israel, Saint Vincent and the Grenadines, United States Minor Outlying Islands, Montenegro, Seychelles, Solomon Islands, Tonga, Angola, Cape Verde, Equatorial Guinea, Kenya, Cuba, Gambia, Zambia, Mauritania, Albania, Kosovo, Serbia, Mauritius, and North Macedonia.

Elizabeth Stifel was responsible for the policies of the following countries: Ethiopia, Eritrea, Madagascar, Sri Lanka, Mozambique, Myanmar, South Africa, Grenada, Jamaica, Kiribati, Maldives, Saint Helena, Ascension and Tristan da Cunha, Bahamas, Bahrain, East Timor, Fiji, Tunisia, Wallis and Futuna, Guinea-Bissau, Sao Tome and Principe, Burundi, Burkina Faso, French Guiana, French Polynesia, Viet Nam, Reunion, Benin, Togo, Senegal, Cameroon, France, Comoros, Chad, Saint Pierre and Miquelon, Saint Barthélemy, Sudan, Ghana, and Saint Kitts and Nevis.

Erin Straight was responsible for Guatemala, Brazil, Paraguay, Mexico, Portugal, United States, Moldova, Ukraine, Kazakhstan, Kyrgyzstan, Afghanistan, Bosnia and Herzegovina, Bulgaria, Belarus, Kuwait, United States, Chile, Tajikistan, Georgia, Namibia, Aruba, Curaçao, and Christmas Island.

Dongying Tao covered the policies of the following countries: China, Hong Kong, Macau, Malaysia, Taiwan, Singapore, Tibet, Botswana, Lesotho, Brunei Darussalam, Mongolia, Pakistan, Malta, Palau, Marshall islands, Papua New Guinea, Niue, Liberia, Zimbabwe, South Sudan, Western Sahara, Somaliland, South Africa, Uganda, Japan, Philippines, Morocco, Slovakia, Vanuatu, and Norfolk Island.

Erin Tutaj was responsible for the policies of Peru, Panama, Costa Rica, El Salvador, Honduras, Nicaragua, Venezuela, Ecuador, Bolivia, Guernsey, Gibraltar, Anguilla, Bermuda, British Indian Territory, Cayman Islands, Antigua and Barbuda, Cyprus, Dominican Republic, Poland, Vatican City, Norway, Slovenia, Hungary, Czechia, Greece, Romania, Nauru, Congo, Comoros, Chad, Burundi, and Burkina Faso.

Mark A. Weiss covered Russia, North Korea, Yemen, Central African Republic, Eritrea, India, Benin, Somalia, Malawi, Sudan, Ghana, Falkland Islands, Pitcairn (Islands), Latvia, Equatorial Guinea, Djibouti, and the Democratic Republic of the Congo.

3 RA Contributions to the Data Review Process

H. A. J. reviewed the policies for: Peru, Panama, Costa Rica, El Salvador, Honduras, Nicaragua, Venezuela, Ecuador, Bolivia, Guernsey, Gibraltar, Anguilla, Bermuda, British Indian Territory, Cayman Islands, Antigua and Barbuda, Cyprus, Dominican Republic, Poland, Vatican City, Norway, Slovenia, Hungary, Czechia, Greece, Romania, Nauru.

E. B. reviewed China, Hong Kong, Macau, Malaysia, Taiwan, Singapore, Tibet, Botswana, Lesotho, Brunei Darussalam, Mongolia, Pakistan, Malta, Palau, Marshall islands, Papua New Guinea, Niue, Liberia, Zimbabwe, South Sudan, Western Sahara, Somaliland, South Africa, Uganda, Japan, Philippines, Morocco, Slovakia, Vanuatu, Norfolk Island.

J. F. reviewed Guadaloupe, Democratic Republic of the Congo, Congo, Barbados, Cook Islands, Saint Martin (French part), Croatia, Sweden, Latvia, Armenia, Spain, Argentina, Uruguay, Colombia, Israel, Saint Vincent and the Grenadines, United States Minor Outlying Islands, Montenegro, Seychelles, Solomon Islands, Tonga, Angola, Cape Verde, Equatorial Guinea, Kenya, Cuba, Gambia, Zambia.

L. F. reviewed Mauritania, Albania, Kosovo, Serbia, Mauritius, North Macedonia, Mali, Guinea, Gabon, Djibouti, Iceland, Turkmenistan, Canada, Australia, Egypt, Nigeria, Sierra Leone, Croatia, Palestine.

C. H. reviewed Ethiopia, Eritrea, Madagascar, Sri Lanka, Mozambique, Myanmar, South Africa, Grenada, Jamaica, Kiribati, Maldives, Saint Helena, Ascension and Tristan da Cunha, Bahamas, Bahrain, East Timor, Fiji, Tunisia, Wallis and Futuna, Guinea-Bissau, Sao Tome and Principe, Burundi, Burkina Faso, French Guiana, French Polynesia, Viet Nam, Reunion, Benin, Togo, Senegal, Nigeria, Cameroon, France, Comoros, Chad, Saint Pierre and Miquelon, Saint Barthélemy, Sudan, Ghana, Saint Kitts Nevis.

N. M. reviewed Guatemala, Brazil, Paraguay, Mexico, Portugal, United States, Moldova, Ukraine, Kazakhstan, Kyrgyzstan, Afghanistan, Bosnia and Herzegovina, Bulgaria, Belarus, Kuwait, United States, Chile, Tajikistan, Georgia, Namibia, Aruba, Curaçao, and Christmas Island.

Elizabeth S. reviewed the United Kingdom, Dominica, Haiti, Saint Lucia, New Caledonia, Thailand, Lao, Cambodia, Indonesia, Bangladesh, Bhutan, Mongolia, Rwanda, Niger, Mayotte, Swaziland, EU/Schengen, Germany, Austria, Liechtenstein, Switzerland, Luxembourg, the Netherlands, Belgium, Lithuania, Finland, Denmark, Estonia, San Marino, Monaco, Italy, Ireland.

Erin S. reviewed Turkey, Greenland, American Samoa, Cocos/Keeling, Faroe Islands, Martinique, Trinidad and Tobago, Tuvalu, Samoa, Tokelau, South Korea, Uzbekistan, Belize, Saudi Arabia, Jordan, UAE, Oman, Qatar, Syria, Nepal, Iran, and Lebanon.

D. T. reviewed Russia, North Korea, Yemen, Central African Republic, Eritrea, India, Benin, Somalia, Malawi, Sudan, Ghana, Falkland Islands, Pitcairn (Islands), Latvia, Guyana, Libya, New Zealand, Tanzania.

E. T. reviewed the Falkland Islands, Pitcairn (Islands), Isle of Man, Jersey, Montserrat, Turks and Caicos Islands, Virgin Islands (British), Sint Maarten (Dutch part), Northern Mariana Islands, Antarctica, Andorra, Bouvet Islands, Puerto Rico, Guam, Svalbard and Jan Mayen, U.S. Virgin Islands, Aland Islands, Bonaire, the Federated States of Micronesia.

M. A. W. reviewed Guadaloupe, Democratic Republic of the Congo, Congo, Barbados, Cook Islands, Saint Martin (French part), Croatia, Sweden, Latvia, Armenia, Azerbaijan, Spain, Argentina, Uruguay, Colombia, Israel, Saint Vincent and the Grenadines, United States Minor Outlying Islands, Montenegro, Seychelles, Solomon Islands, Tonga, Angola, Cape Verde, Equatorial Guinea, Kenya, Cuba, Gambia, Zambia, Algeria.

4 Islands Only: Effects of Domestic Lockdowns

We tested the effects of domestic lockdowns on the rate of new cases per capita (IHST) for only the island countries. The matching process yields 39 matched sets out of a total of 51 treatment observations. The refinement strategy of the estimates shown in green is CBPS. The effects as seen in Figure 1 are similar to those observed when testing the full set of countries, though with much larger confidence intervals. Again we observe statistically significant positive estimates during the pre-analysis period, an negative estimates, though not statistically significant from T_{+2} to T_{+5}.

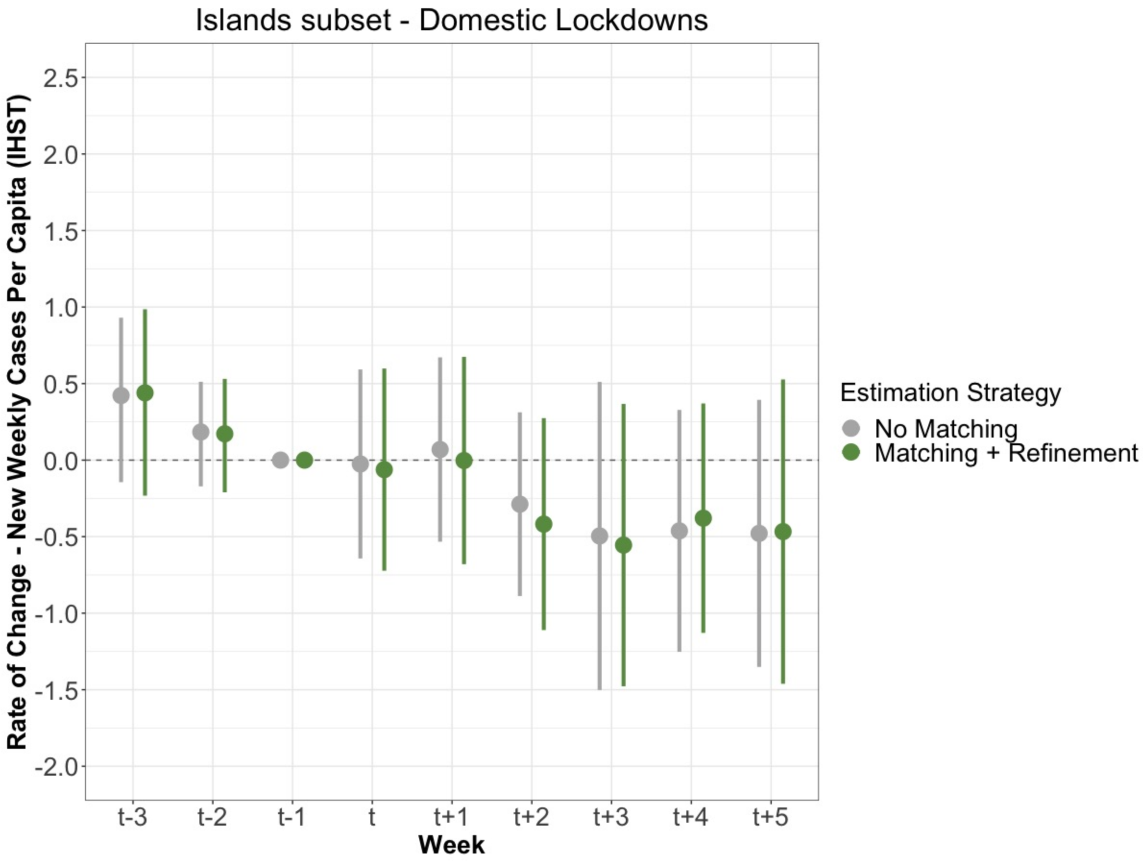

Figure 1: The measured effects of domestic lockdown on the rate of change of new cases per capita IHST (y-axis) for a sub-set of 89 island nations. The estimates shown in grey were calculated with both no matching or refinement, while the estimates in green were calculated using both matching and refinement, both shown with 95% confidence intervals. The period includes nine weeks, three prior to the lockdown, the week of the lockdown, and five following the lockdown.

5 Construction and Analysis of Dependent Variable

This paper seeks to measure whether SARS-CoV-2 was meaningfully impacted by large-scaled policy changes. Below we present normality tests for three different variations on our dependent variable and discuss our theoretical approach to variable construction. Our final selection of the dependent variable is based upon both theoretical and statistical concerns.

Below are normality test plots calculated using the *LambertW* package in R, showing an Autocorrelation Function (ACF) test, Density Plot with Skewness and Kurtosis measures, and a Quantile-Quantile (QQ)-pplot. A normal distribution has a skewness of 0 and kurtosis of 3.

First, we recognize the importance of measuring new cases per capita, as country populations vary dramatically in size. Figure 2 shows the normality tests for the number of new SARS-CoV-2 cases in a specific week and then divided by the country population, per 100,000 residents. All of the normality test point to significant imbalance in the upper quantiles, given a handful of observations with very high values. For example, this variable has the mean is 45.6, minimum of 0, and maximum is 1199.8.

We deem this measure as insufficient from both theoretical and statistical standpoints. Policy interventions should be more directly related not to the number of new cases, but the rate of change of new cases. Thus, the measure rate of change for new cases per capita takes the population adjusted measure and subtracts it from the value of the previous week. Figure 3 presents the normality tests for this variable which is closely centered around zero, indicating no or very little change in new SARS-CoV-2 rates after population adjustment for most observations. Our tests indicate that this variables demonstrates some skewness (0.82), and extremely high kurtosis (183) with heavy (or long) tails in both directions. This generated significant concern that a few prominent outliers were driving our main results.

To help reduce kurtosis, we applied an Inverse Hyperbolic Sine Transformation (IHST) to our rate of change in new cases per capita variable. [**?**] The normality test results are shown in Figure 4. This variable is not completely balanced or normal, but reports a low skewness of -0.026, and much improved kurtosis of 3.5, fairly similar to a normal distribution. Because of this significant improvement in normality and balance, we rely on the IHST version while testing.

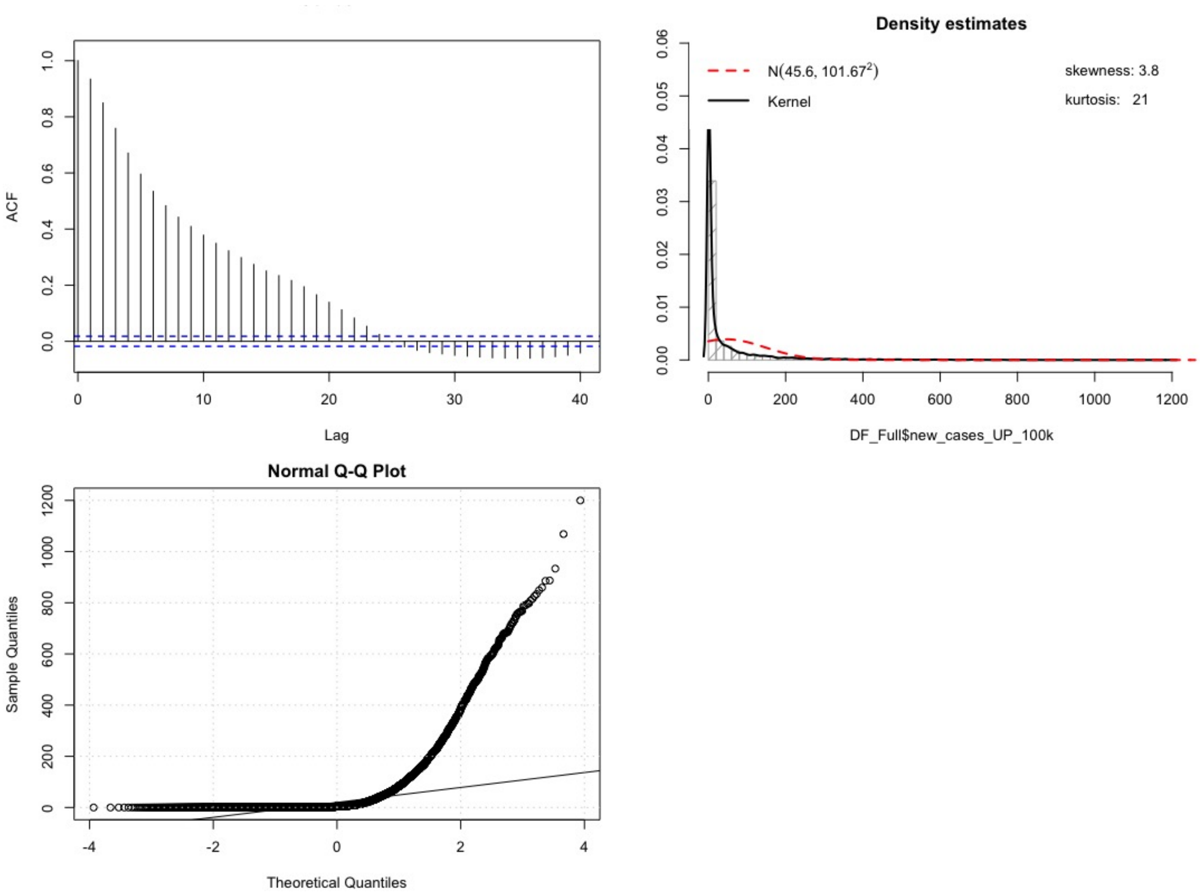

Figure 2: Normality tests of the variable number of new SARS-CoV-2 cases per country-week after controlling for country population size. The three plots assess autocorrelation, density, and quantile balancing.

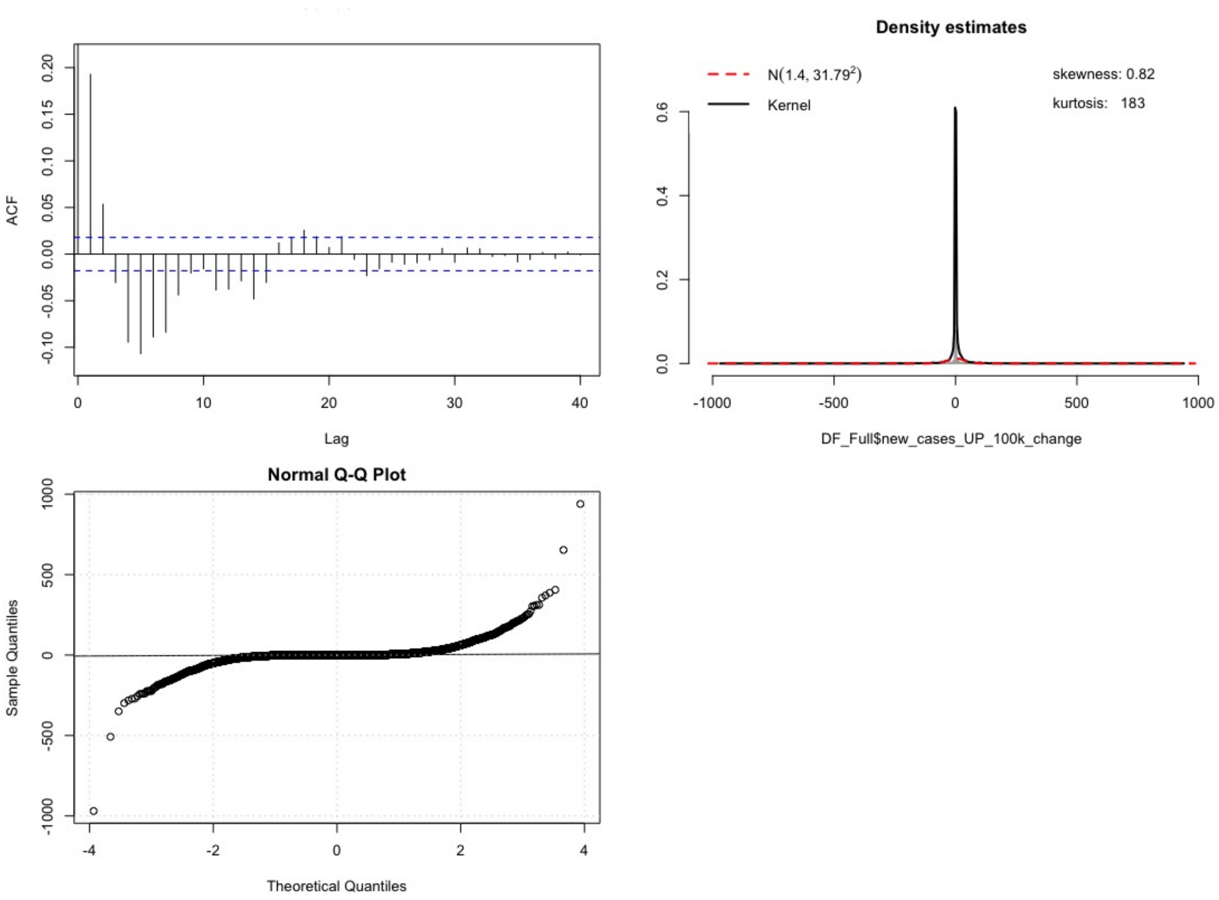

Figure 3: Normality tests of the variable rate of change of new SARS-CoV-2 cases per country-week after controlling for country population size. The three plots assess autocorrelation, density, and quantile balancing.

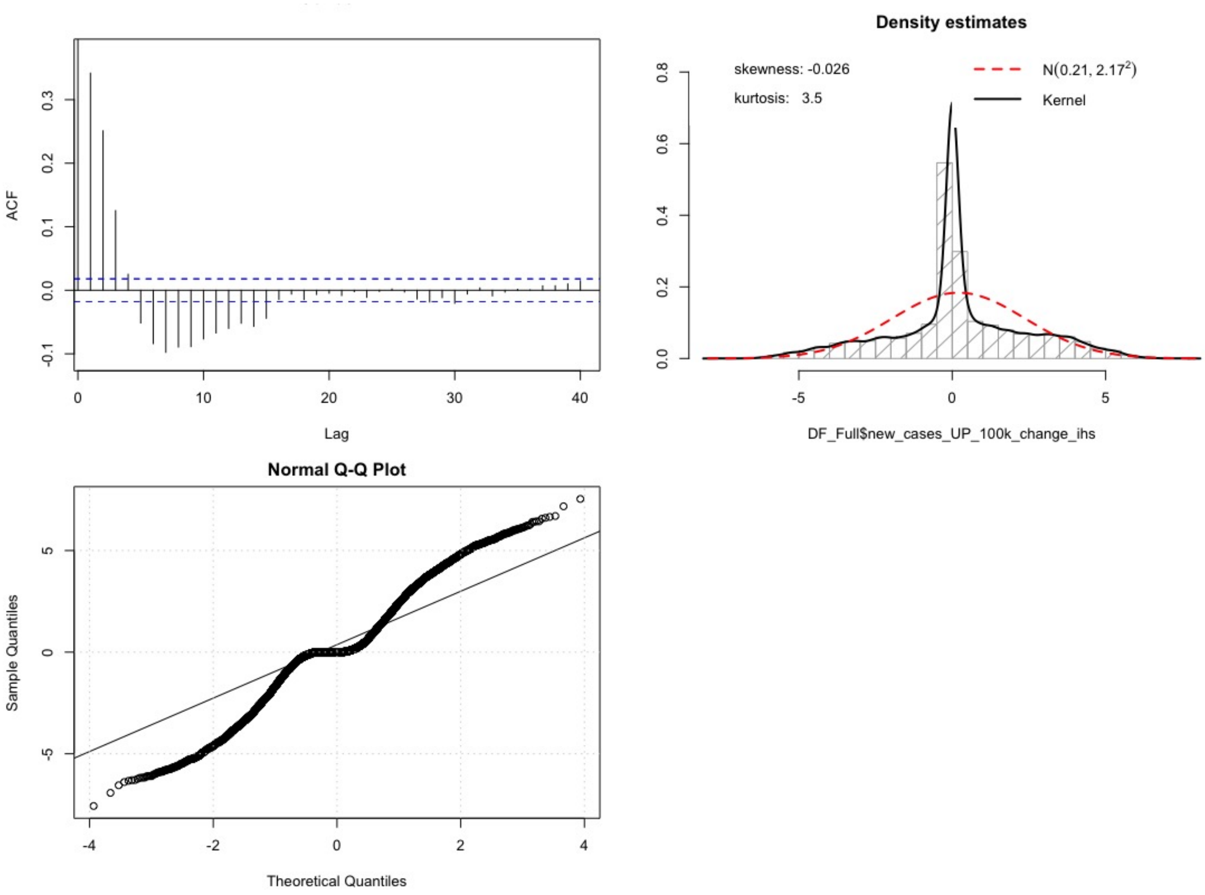

Figure 4: Normality tests of the variable rate of change of new SARS-CoV-2 cases per country-week after controlling for country population size and undergoing an inverse hypobolic sine transformation. The three plots assess autocorrelation, density, and quantile balancing.

6 Covariate Balancing Analysis

Our inference relies on the assumption that control and treatment units are similar across a core set of covariates. In the case of the panel matching model, covariate balance is assessed during each of the pre-treatment and treatment periods by "taking the average of the difference between the values of the specified covariates for the treated unit(s) and the weighted average of the control units across all matched sets." [?] The results are expressed in standard deviations for each weekly period. The key threshold used for determining "good" balance is typically 0.20 or less. In the analyses below, we show all covariate differences as absolute values due to aggregation.

The figures below demonstrate the average standardized mean differences across time period, variable type, and refinement strategy. The four refinements strategies examined are None, Mahalanobis, Propensity Score (PS), and Covariate Balancing Propensensity Score (CBPS). The model variables are also disaggregated into two categories: fixed across time and varying week to week. The fixed variable category includes: GDP Per Capita (log), Human Development Index, Liberal Democracy, Free Expression and Assess to Information, Population 2020 (log), Percent Population over 70, Land Area (log), Median Population Age, and Average Life Expectancy. The time-varing variables are: New Cases Per Capita Rate of Change (IHST), Daily Tests Per Capita, Stringency Index, and the Treatment variables of Complete Closure, Partial Closure, and Domestic Lockdown. Vaccine-related variables are also included in the model, but do not demonstrate enough variation to be included in the covariate balancing calculation.

Figure 5 presents the balancing statistics for the complete closures analysis. In our main analysis, we select PS as the optimal refinement strategy given its improvement in balancing, demonstrating the lowest standardized mean differences. The average for both fixed and time-variant groups are below the 0.20 threshold for all time periods when PS refinements is applied, generating significantly better balance than if no refinement were applied.

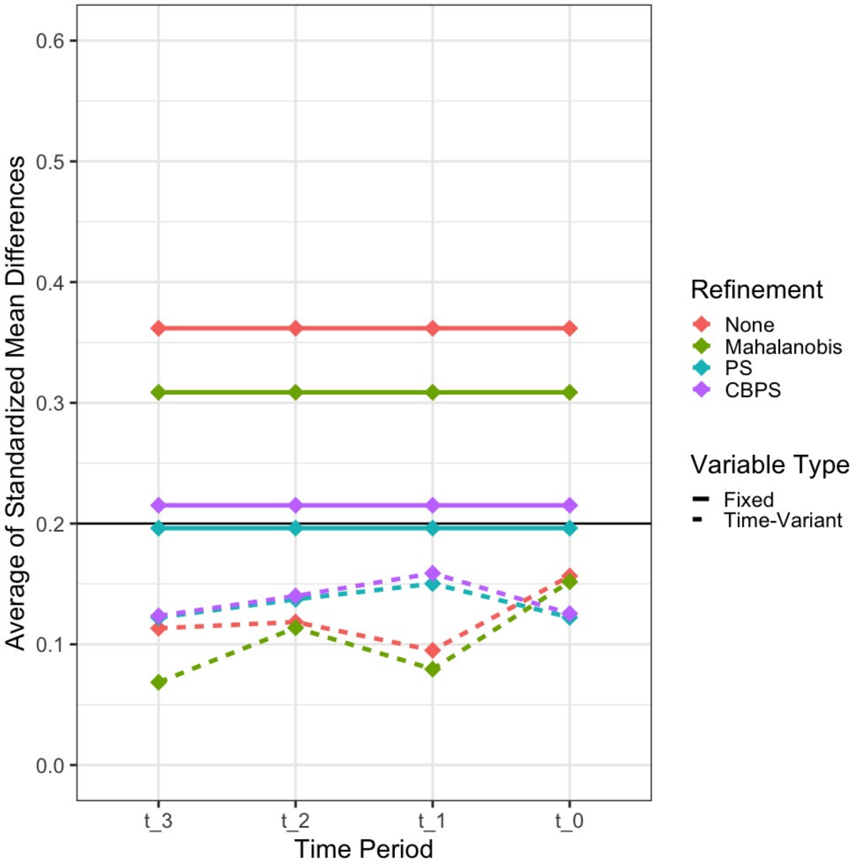

Figure 5: Covariate balancing analysis for the complete closures model. All model variables are sorted into time varying or fixed groups and their average standardized mean differences are shown across the three week prior to treatment and week of treatment. Four refinement strategies are compared, and the lowest (most balanced) is selected for analysis in the manuscript.

Figure 6 shows how refinement influences balancing when examining the model for partial closures. In this case, both PS and CBPS are essentially identical and generate a much more balanced covariate structure in the pre-analysis period compared to not implementing any refinement strategy. Critically, across both PS and CBPS, all the variable group averages are below the 0.20 threshold.

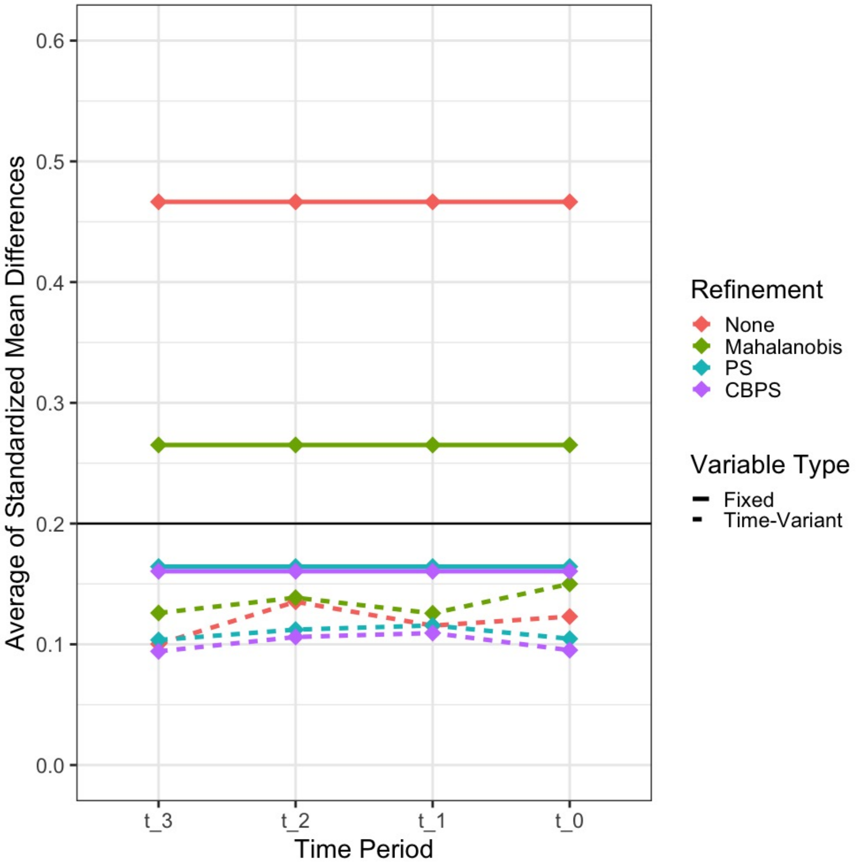

Figure 6: Covariate balancing analysis for the partial closures model. All model variables are sorted into time varying or fixed group sand their average standardized mean differences are shown across the three week prior to treatment and week of treatment. Four refinement strategies are compared, and the lowest (most balanced) is selected for analysis in the manuscript.

Figure 7 presents the refinement results for the domestic lockdown model. In comparison to the border closure policies, refinement strategies are not able to improve balance in the pre-analysis period to the, though CBPS generates the most balanced structure. While the fixed time variables are responsive to refinement strategies, time-varying variables are quite imbalanced, particularly at T_0 the week to treatment. This is also why the pre-treatment periods for the main lockdown results are positive and statistically significant.

During the week of treatment – the mean differences are much higher for the dependent variable of rate of change of new cases per capita Rate (IHST) is 0.40, daily tests per capita is 0.17, partial closures is 0.16. The imbalance on our dependent variables suggests that it is both a cause and effect of domestic lockdowns, which is problematic from a causal inference standpoint, but logical in the real world context. We should expect that rising case rates prompts policy action, which the subsequently results in lower cases rates if effective, which is the pattern observed for domestic lockdowns.

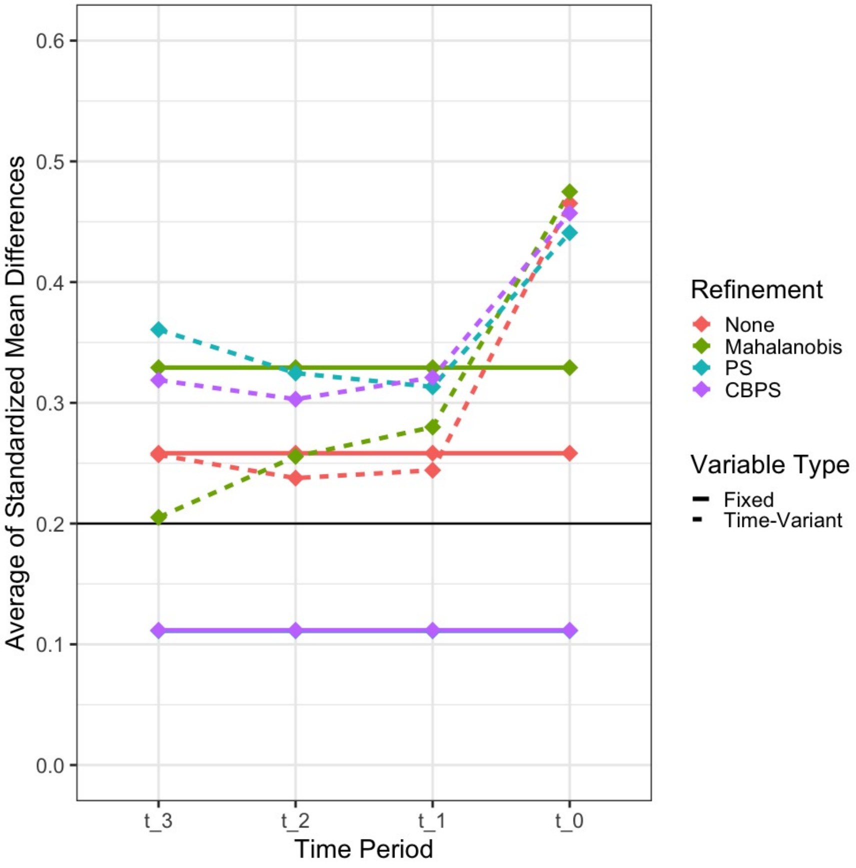

Figure 7: Covariate balancing analysis for the domestic lockdown model. All model variables are sorted into time varying or fixed groups and their average standardized mean differences are shown across the three week prior to treatment and week of treatment. Four refinement strategies are compared, and the lowest (most balanced) is selected for analysis in the manuscript.

7 Average Balancing by Policy and Refinement Strategy

Table 1 presents the degree of average imbalance across all policies tested int he manuscript and the available refinement strategies. These figures guide our selection of which refinement strategy to apply in the model for each treatment variable. The value for each policy-refinement combination represents all covariates at T_{-1}, the week used as the difference-in-difference baseline.

The value presented for each model imbalance calculated as follows. First, the average of standardize means differences are calculated for each treatment variable and refinement method including "None-None", representing neither matching nor refinement as a baseline for improvement. The standardized mean different is selected for each variable at T_{-1}, and their absolute values over 0.20 are average across all relevant covariates. This results in a comparable measure across models, where 0 represents all variables falling within the -0.20 to 0.20 range implying excellent balance in the pre-treatment period. The refinement methods with the lowest imbalance score is selected as the modeling strategy in the manuscript.

**Policy Intervention**	**None-None**	**M-Mahal**	**M-PS**	**M-CBPS**	**Selection**
OxGRT Domestic Lockdowns	0.088	0.120	0.053	0.050	CBPS
Lockdowns Islands (Subset)	0.188	0.110	0.143	0.124	Mahal
**COBAP Complete Closures**	0.128	0.076	0.034	0.048	PS
Specific Country	0.556	0.195	0.039	0.044	PS
Work Exception	0.293	0.128	0.021	0.010	CBPS
Citizen Exception	0.059	0.058	0.029	0.049	PS
Essentials Only	0.109	0.039	0.043	0.028	CBPS
Islands (Subset)	0.128	0.107	0.116	0.112	Mahal
**COBAP Partial Closures**	0.246	0.068	0.009	0.006	CBPS
Visa Ban	0.072	0.051	0.006	0.001	CBPS
Citizenship Ban	0.368	0.168	0.029	0.063	PS
Travel History Ban	0.244	0.115	0.013	0.013	CBPS
Border Closures	0.127	0.040	0.002	0.004	PS
Islands (Subset)	0.364	0.334	0.289	0.296	PS

Table 1: Comparison of imbalance across all treatment variables and refinement methods for T_{-1}. The value represents a general measure of model imbalance across all covariates, and the refinement methods with the lowest imbalance score is selected as the modeling strategy in the manuscript.
